# Regulatory T Cell Induction during *Plasmodium chabaudi* Infection Modifies the Clinical Course of Experimental Autoimmune Encephalomyelitis

**DOI:** 10.1371/journal.pone.0017849

**Published:** 2011-03-25

**Authors:** Alessandro S. Farias, Rafael L. Talaisys, Yara C. Blanco, Stefanie C. P. Lopes, Ana Leda F. Longhini, Fernando Pradella, Leonilda M. B. Santos, Fabio T. M. Costa

**Affiliations:** Departmento de Genética, Evolução e Bioagentes, Universidade Estadual de Campinas (UNICAMP), Campinas, São Paulo, Brazil; Agency for Science, Technology and Research - Singapore Immunology Network, Singapore

## Abstract

**Background:**

Experimental autoimmune encephalomyelitis (EAE) is used as an animal model for human multiple sclerosis (MS), which is an inflammatory demyelinating autoimmune disease of the central nervous system characterized by activation of Th1 and/or Th17 cells. Human autoimmune diseases can be either exacerbated or suppressed by infectious agents. Recent studies have shown that regulatory T cells play a crucial role in the escape mechanism of *Plasmodium* spp. both in humans and in experimental models. These cells suppress the Th1 response against the parasite and prevent its elimination. Regulatory T cells have been largely associated with protection or amelioration in several autoimmune diseases, mainly by their capacity to suppress proinflammatory response.

**Methodology/Principal Findings:**

In this study, we verified that CD4^+^CD25^+^ regulatory T cells (T regs) generated during malaria infection (6 days after EAE induction) interfere with the evolution of EAE. We observed a positive correlation between the reduction of EAE clinical symptoms and an increase of parasitemia levels. Suppression of the disease was also accompanied by a decrease in the expression of IL-17 and IFN-γ and increases in the expression of IL-10 and TGF-β1 relative to EAE control mice. The adoptive transfer of CD4^+^CD25^+^ cells from *P. chabaudi*-infected mice reduced the clinical evolution of EAE, confirming the role of these T regs.

**Conclusions/Significance:**

These data corroborate previous findings showing that infections interfere with the prevalence and evolution of autoimmune diseases by inducing regulatory T cells, which regulate EAE in an apparently non-specific manner.

## Introduction

The determinants underlying the heterogeneity of multiple sclerosis (MS) remain unclear. However, current evidence indicates the involvement of a complex genetic trait that probably requires an environmental factor, such as an infection, to be triggered [1]. Classical studies have demonstrated that experimental autoimmune encephalomyelitis (EAE), an experimental model of MS, can be either exacerbated or suppressed by infectious agents [2]–[6]. EAE is an inflammatory demyelinating autoimmune disease of the CNS that is characterized by the activation of Th1 and/or Th17 cells [7], [8]. Amplification of the response of these lymphocytes leads to tissue injury, which can result in demyelination. Regulatory mechanisms might be activated to down regulate exacerbated inflammatory responses. Indeed, regulatory cells that are positive for the expression of the transcription factor Foxp3 have a crucial function in activating immune suppression and in the maintenance of immune homeostasis [9]–[11]. A deficiency in either the number or function of Foxp3-positive T cells has been described in both the MS and the EAE model [12]–[15]. The Foxp3^+^ IL-10-producing cells are associated with the recovery phase of EAE, and *in vitro-*generated or purified natural regulatory T cells prevent the induction of EAE by producing IL-10 [16].

On the other hand, some studies have shown that the activation of T regs, either in experimental models of malaria or in humans infected by the malaria parasite, suppresses the Th1 response and prevents elimination of the parasite. The enhancement of CD4^+^CD25^+^ regulatory T cells probably plays a crucial role in this escape mechanism [17]–[22]. These natural regulatory cells seem to be associated with a burst of TGF-β production and decreases in antigen-specific responses and proinflammatory cytokine production [23].

In the present study, we used experimental malaria infection and the murine MS model to investigate how the regulatory mechanisms induced during *Plasmodium* infection interfere with the clinical course and immune responses in the EAE model.

## Materials and Methods

### Animals

C57BL/6 mice (6–8 weeks old) were purchased from the University of Campinas (Campinas, SP, Brazil) and maintained in a specific pathogen-free animal facility. All experiments and procedures were approved by the UNICAMP Committee for Ethics in Animal Research (Protocol No. 857-1).

### Induction of EAE

Briefly, mice were injected subcutaneously (s.c.) with 100 µg/animal of pMOG_35-55_ (MEVGWYRSPFSRVVHLYRNGK) or pCIR_180-198_ (NPYCNVLTNLKN DYDKIRK) (Genemed Synthesis, CA, USA) emulsified in complete Freund's adjuvant containing 4 mg/ml of *Mycobacterium tuberculosis* H37RA (Difco, Detroit, MI, USA). Each immunized animal receives a total of 100 µl of the emulsion in the both upper flanks and 200 ng/mouse of Pertussis toxin intraperitoneal (i.p.) (List Biochemicals, Campbell, CA, USA) on days 0 and 2 after immunization. Clinical expression of the disease was graded on a clinical index scale of 0 to 5 as previously described [24].

### Malaria infection

Mice were infected i.p. with 10^6^ infected red blood cells (iRBCs) of the non-lethal line of *P. chabaudi chabaudi* AS or were injected with saline only (negative control group), 6 days after EAE induction (6 d.a.i) or 25 days prior the EAE induction (post-infection with *P. chabaudi*). The group injected with *P. chabaudi* 6 d.a.i. presented a peak of parasitemia in the same day of EAE maximum clinical score.

The blood stage forms of both parasites were stored in liquid nitrogen after *in vivo* passages in C57BL/6 mice according to a protocol described elsewhere [25]. The percentage of parasitemia was determined daily by counting the number of iRBCs among at least 1,000 erythrocytes in Giemsa-stained blood smears. The corporal temperatures and relative body weights of the mice were evaluated daily, starting on day 1 post-infection, by rectal introduction of a precision digital thermometer (model TE-300, Instrucamp, Brazil) and with a precision balance (Metter Toledo), respectively.

### Flow Cytometry

All analyses were performed using a flow cytometer (FACScanto or FACSCalibur) (BD Bioscience, San Jose, CA, USA). For Foxp3 labeling, permeabilization buffer (PBS 10% rat serum and 1% Triton) was used. The antibodies were as follows: anti-CD4 FITC, anti-CD4 PE, anti-CD25 PE (BD Bioscience, San Jose, CA, USA) and anti-Foxp3 APC (eBioscience, San Diego, CA, USA). The data were analyzed using FACSDiva (BD Bioscience, San Jose, CA, USA) or MDI 2.9 software.

### Cell sorting

All sorting were performed using a cell sorter flow cytometer FACSAria (BD Bioscience, San Jose, CA, USA). Cells were kept on ice before and after sorting analysis. Cell purity was confirmed immediately after sorting and cell viability was assured before the transfer. Each animal was injected (i.v.) with 1×10^6^ viable cells.

### Quantitative real-time PCR (qRT-PCR)

mRNA was extracted using Trizol and reverse transcribed to generate cDNA. Taqman analysis was performed using a Taqman ABI Prism 7500 Sequence Detector (PE Applied Biosystems, Darmstadt, Germany). The primers GAPDH, IL-17A, IL-10, TGF-β_1_, IL-27 and Foxp3 were obtained from Applied Biosciences (Mm00439619_m1 (IL-17A), Mm99999915_m1 (GAPDH), Mm00475156_m1 (Foxp3), Mm03024053 (TGFβ1), Mm00461164 (IL-27), Mm00439616_m1 (IL-10)). The specific mRNAs were normalized to the expression of a housekeeping gene (GAPDH). The data were obtained using independent duplicate measurements. The threshold cycle value of the individual measurements did not exceed 0.5 amplification cycles.

### Statistical Analysis

The statistical significance of the results was determined using a non-parametric analysis of variance (Kruskal-Wallis test), the Mann-Whitney test (U-test) or a non-parametric correlation (Spearman's rank). A *p* value less than 0.05 was considered significant.

## Results

### Modulation of EAE and P. chabaudi infection

**Figure 1:**
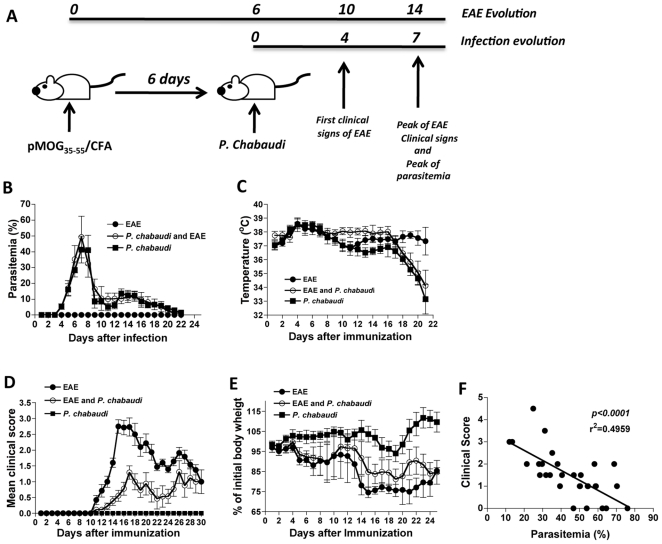
Malaria infection *P. chabaudi*-infected mice 6 d.a.i ameliorates clinical signs of autoimmunity. (**A**) Experimental design of EAE induction and *P. chabaudi* infection. (**B**) Parasitemia levels, (**C**) corporal temperature, (**D**) EAE clinical score and (**E**) weight loss in groups of mice infected with *P. chabaudi* alone (*P. chabaudi* - black squares), *P. chabaudi* infected 6 days after EAE induction (*P. chabaudi* + EAE - white circles), and in mice harboring EAE alone (EAE - black circles). The results are expressed as the mean of each group of mice (n = 5–8) ± SD. (**F**) Correlation between parasitemia level and EAE clinical score in the pool of mice harboring a malaria infection 6 days after EAE induction.

The inductions of EAE and *P. chabaudi* infection were designed to coincide with the peak of the clinical symptoms of EAE. EAE was more severe approximately 14 days after immunization with pMOG_35-55_, and the peak of parasitemia occurred 7 days after infection (Fig. 1A). The presence of an autoimmune response during EAE induction did not change the parasitemia levels (Fig. 1B) or the corporal temperature of the animals during the course of malaria infection (Fig. 1C). However, the clinical course of EAE was significantly diminished until 28 days post-EAE induction (Fig. 1D). The reduction in clinical signs correlated with a loss of body weight. As shown in Figure 1E, animals with EAE and infected with *P. chabaudi* 6 d.a.i presented a modest loss of body weight relative to the group with EAE alone. Of note, parasitemia and clinical symptoms of EAE were significantly negatively correlated during the clinical course of the disease in the group of mice infected with *P. chabaudi* 6 d.a.i (Fig. 1F).

**Figure 2:**
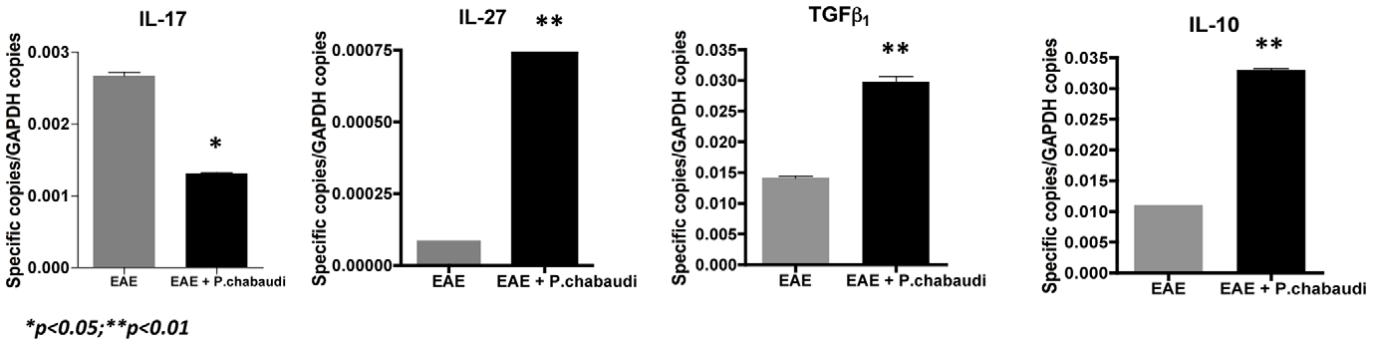
Induction of EAE in *Plasmodium chabaudi*-infected mice modulates their cytokine expression profile. Expression levels of IL-17A, IL-10, IL-27 and TGF-β_1_ measured by qRT-PCR in *P. chabaudi* infected 6 days after EAE induction (black bars) and the EAE alone (gray bars) group. The values represent the mean number of specific cytokine gene copies relative to GAPDH in three-five mice ± SD.

Next, we measured cytokine expression by qRT-PCR to verify whether the reduction in the clinical signs of EAE observed in infected with *P. chabaudi* 6 d.a.i was related to differences in the cytokine expression profile. As shown in Figure 2, a significant reduction in IL-17A expression was noted in the regional lymph nodes in the EAE-malaria group in comparison to the EAE group. Moreover, corroborating the reduction in the pro-inflammatory profile, we discovered higher expression of IL-10, TGF-β_1_ and IL-27 in both lymph nodes in the EAE-malaria mice relative to the EAE-alone mice (Fig. 2).

### Involvement of regulatory T cells

**Figure 3:**
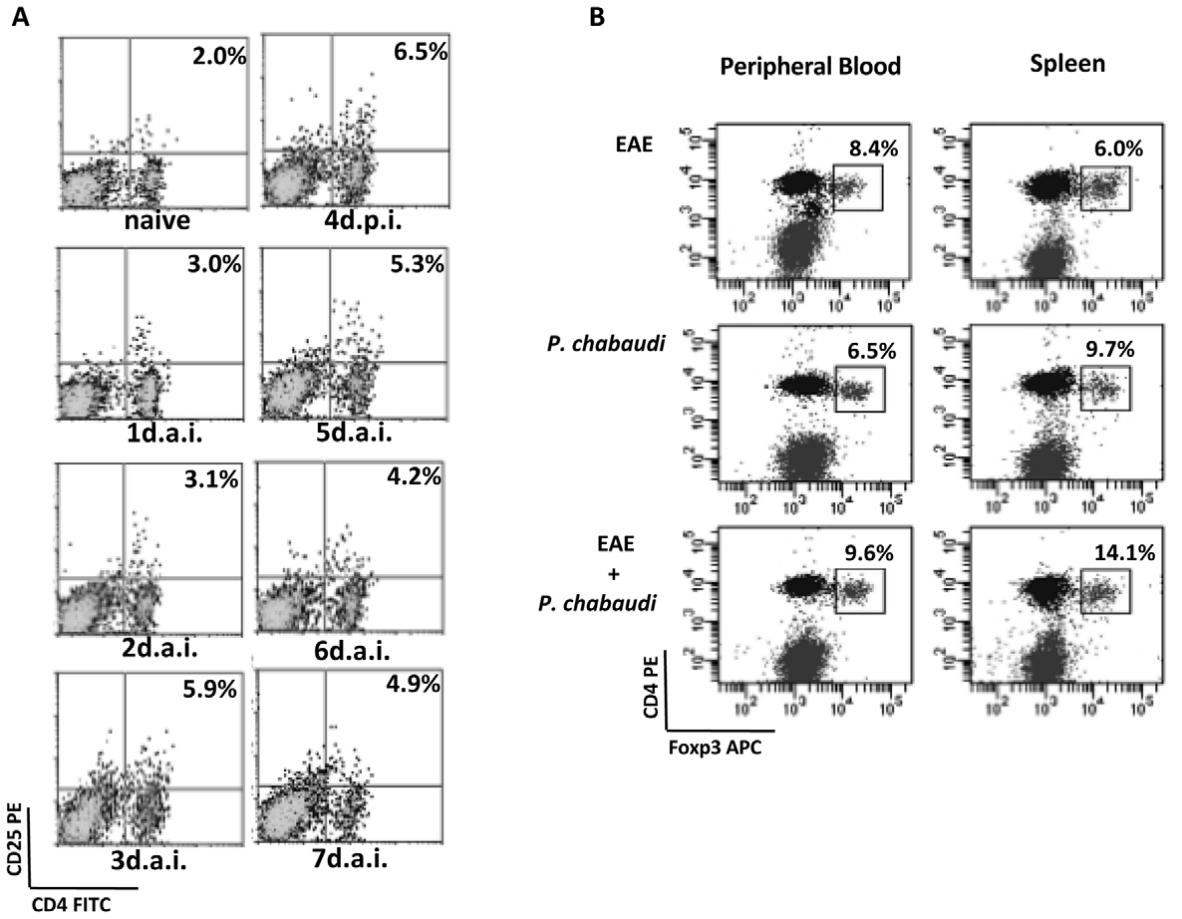
Mice harboring a malaria infection display an increase in the percentage of T regs during EAE evolution. (**A**) Daily quantification of CD4^+^CD25^+^ cells in *P. chabaudi*-infected mice on days 0–7 post-infection. (**B**) Quantification of CD4^+^CD25^+^Foxp3^+^ cells 4 days post-infection and/or 10 days after EAE induction. The percentages are relative to the total number of CD4^+^ cells and are representative of three independent assays.

Corroborating with previous studies investigating experimental malaria infection models [17], the percentages of CD4^+^CD25^+^ regulatory T cells increased in the spleen as the parasitemia evolved, with a peak observed on day 4 after infection (6.4%) (Fig. 3A). By contrast, naïve mice presented CD4^+^CD25^+^ percentages (2.0%) were three fold lower than those detected in malaria-infected mice. A significant difference in the percentage of T regs was noted on day 4 post-infection in the spleen of EAE mice when compared to EAE-malaria-infected animals (Fig. 3B). Indeed, whereas only 6.0% of the cells harvested from EAE mice were CD4^+^CD25^+^Foxp3^+^ T cells, in the EAE animals harboring a malaria infection, up to 14.1% of the CD4+ cells expressed CD4^+^CD25^+^Foxp3^+^. Analyses of peripheral blood cells revealed a minor increase in the percentage of T reg cells in the EAE-malaria (9.6%) relative to the EAE-only mice (8.4%). Nevertheless, these values did not reach statistical significance (Fig. 3C).

### CD4^+^CD25^+^ regulatory T cell transfer

**Figure 4:**
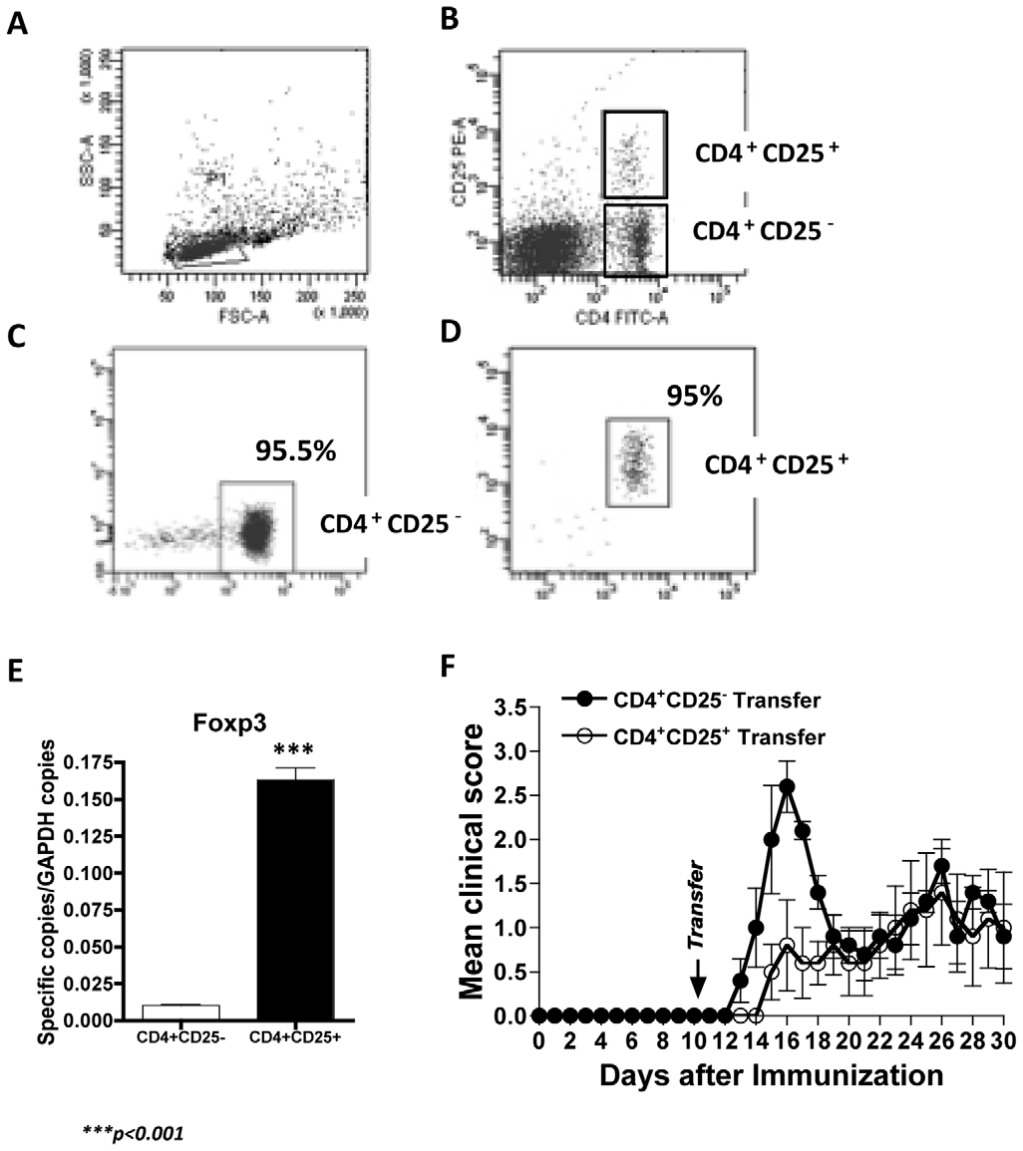
Transfer of CD4^+^CD25^+^ regulatory T cells diminishes the clinical signs of EAE. (**A**) Lymphocytes are gated from whole spleen cells from a pool of 3–4 mice infected with *P. chabaudi*. (**B**) Selection of CD4^+^CD25^+^ and CD4^+^CD25^−^ cells before sorting. (**C**) Purity of the sorted CD4^+^CD25^−^ and (**D**) CD4^+^CD25^+^ cells. (**E**) qRT-PCR assessment of expression levels of Foxp3 in CD4^+^CD25^+^ and CD4^+^CD25^−^ cells after sorting. The values represent the mean number of specific cytokine gene copies relative to GAPDH ± SD. **(F**) Evolution of the clinical scores of EAE following the transfer of CD4^+^CD25^+^ (white circles) or CD4^+^CD25^−^ cells (black circles). The results represent the mean of three-five mice ± SD.

To verify the participation of CD4^+^CD25^+^ cells in the regulation of autoimmune responses during EAE, we transferred purified CD4^+^CD25^+^ T cells from *P. chabaudi*-infected mice four days after infection into MOG_35-55-_immunized mice. No parasites were detected in the CD4^+^CD25^+^ cell preparation. Sorting analyses revealed that CD4^+^CD25^−^ (Fig. 4C) and CD4^+^CD25^+^ (Fig. 4D) T cells were highly pure (95%±2%) and expressed high levels of Foxp3, as revealed by testing an aliquot of 10^6^ sorted cells (Fig. 4E). Thus, each set (CD4^+^CD25^−^ or CD4^+^CD25^+^) of T cells were transferred intravenously into a different group of MOG35-55 immunized mice 10 days after EAE induction. The transfer of CD4^+^CD25^+^ cells harvested and sorted from EAE-malaria mice significantly diminished the evolution of clinical signs of EAE (*p*<0.01) as compared to animals that were immunized only, or even to the group that received CD4^+^CD25^−^ cells (Fig. 4F). Of note, the anti-inflammatory effect of the T regs was temporary, because 10 days after the transfer, no significant difference in EAE clinical scores were found between the two groups.

### Longevity of the regulatory T cell effect

**Figure 5:**
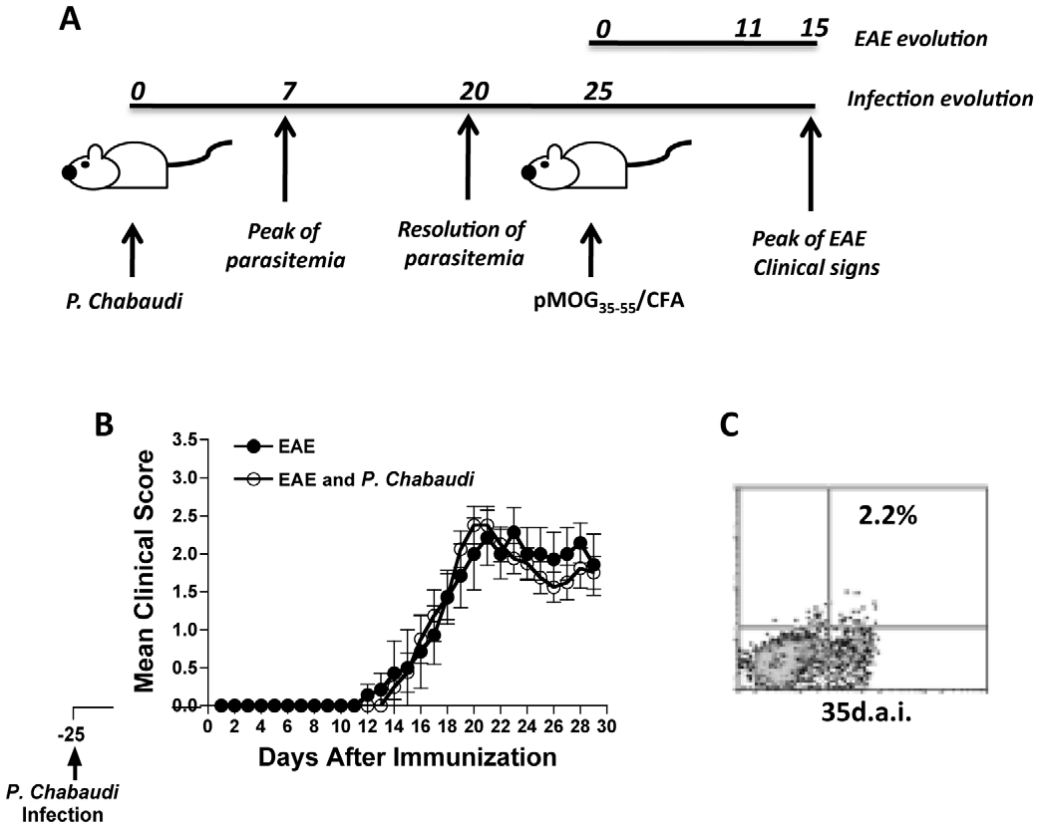
Induction of EAE after recovery from malaria infection does not reduce clinical signs. (**A**) Experimental design of post *P. chabaudi* infection EAE induction in *P. chabaudi*-infected mice (n = 5–7). (**B**) EAE induction 25 days post-*P. chabaudi* infection (white circles) or EAE induction alone (black circles). The results represent the mean of three-five mice ± SD. (**C**) CD4^+^CD25^+^ quantification 25 days post-*P. chabaudi* infection.

To determine whether the malaria-induced regulatory T cells were able to sustain their anti-inflammatory effect and thereby prevent mice from developing clinical signs of EAE, we induced EAE 25 days after infection with *P. chabaudi* (Fig. 5A). At this time point, almost no parasites were detected in the peripheral blood (Fig. 1B). No significant differences in the evolution of the disease were observed between the group with previous *P. chabaudi* infection and the group that was immunized with MOG peptide only (EAE) (Fig. 5B). Indeed, this observation is consistent with the low percentage of CD4^+^CD25^+^ T cells (2.2%) harvested from the mice 35 days p.i. (Fig. 5C), which did not differ significantly from the percentage observed in naïve animals. These results indicate that the mechanism that suppresses the autoimmune response in *P. chabaudi* infection is transitory and does not generate a specific memory.

## Discussion

In the present study, we used *P. chabaudi* infection and EAE induction to demonstrate that the immune response to *Plasmodium* spp. modulates the autoimmune response.

One of the main challenges of multiple sclerosis is an understanding its etiology. It is currently well accepted that the disease results from a genetic predisposition combined with an environmental factor such as an infection [26]. However, many recent studies have demonstrated an immunoregulatory role of infectious agents or their products during the evolution of EAE or MS [4]–[6]. In all likelihood, both observations are compatible depending on the genetic predisposition of each individual, the specific action of each infectious agent and the specific timing of the infection.

Here, we conducted a study to understand the effects of malaria infection on the evolution of MS in a murine experimental model. When EAE induction coincided with the peak of infection in *P. chabaudi*-infected mice, we observed a reduction in the clinical signs of EAE, including weight loss. Of note, we discovered a negative correlation between the clinical score for EAE and the percentage of iRBCs. The protective effect of malaria infection on EAE evolution was correlated with the high expression levels of IL-10, IL-27 and TGF-β_1_ and reduced levels of IL-17. Moreover, an elevated percentage of T regs on day 4 p.i. with *P. chabaudi* seemed to play a pivotal role in EAE amelioration. Interestingly, the presence of a proinflammatory response evoked during EAE evolution did not alter the evolution of parasitemia. The specific proinflammatory response to MOG_35-55_ might not influence reactivity to malarial antigens. The suppression of immune responses by *Plasmodium* parasites might be explained, at least in part, by the capacity of human or murine iRBCs to convert latent TGF-β into its bioactive form [27]. The activation of TGF-β by the parasite might induce the conversion of naïve T cells into T reg cells, or it might directly suppress the autoimmune response [28]–[29].

In recent years, a growing body of evidence has demonstrated the importance of T regs in the immunological response induced during malaria infection in humans [19]–[22], and in several experimental models [17], [30]. The enhancement of T regs apparently contributes to the immune evasion mechanism of *Plasmodium spp.* and allows parasite development, although this phenomenon may not hold true for all murine-derived *Plasmodium* species [31]. In vitro co-cultures of PBMCs and *P. falciparum*-infected red blood cells induce CD4^+^CD25^+^Foxp3^+^ T cells [32], and a positive correlation between the absolute number of circulating T regs and the parasite burden during acute *P. vivax* infection has been recently observed [22].

Our data demonstrate a progressive increase in the percentage of CD4^+^CD25^+^ T cells during *P. chabaudi* infection, with a peak observed on day 4 after infection. These CD4^+^CD25^+^ T cells express high amounts of Foxp3 in both *P. chabaudi*-infected mice and in *P. chabaudi*-infected mice 6 d.a.i when compared with EAE-alone mice. This finding may explain the observed suppressive effect of malaria infection on the course of EAE. A large number of reports have highlighted the role of regulatory T cells both in the prevention of EAE induction [33]–[34] and in the resolution of the disease [16], [35], mainly via IL-10 and/or TGF-β induction in these cells [36].

Because CD4^+^CD25^+^Foxp3^+^ T regs are capable of inhibiting proinflammatory responses by releasing IL-10 and TGF-β_1_, in our model and in other autoimmune models [10], it is conceivable that these CD4^+^CD25^+^Foxp3^+^ T regs might play a central role in regulating the autoimmune response during *Plasmodium* infection. To test this hypothesis, we transferred highly pure sorted CD4^+^CD25^+^ T cells from *P. chabaudi*-infected mice four days after infection into MOG_35-55_ immunized mice, avoiding the introduction of parasites into the system. The adoptive transfer of CD4^+^CD25^+^ T cells, which express higher amounts of Foxp3, but not of CD4^+^CD25^−^ cells, significantly diminished the clinical signs of EAE for approximately 10 days, similar to the signs observed with *P. chabaudi*-infected mice 6 d.a.i. These results unequivocally demonstrated the central role of malaria-induced T regs in the control of EAE development. However, when EAE was induced in a group of mice with controlled parasitemia, no amelioration of the clinical signs of EAE was observed, and the percentage of CD4^+^CD25^+^ cells was comparable to that in naïve animals. Our findings indicate that the process of T reg cell enhancement is not long lasting and probably does not induce immunological memory. Indeed, these T regs are rapidly inducible and short-lived, as previously described [37]. Nonetheless, malaria-induced T regs are able to suppress responses to non-malarial antigens [38].

Despite these indications, we tried to establish some cross-specificity between the MOG protein and *P. chabaudi* antigens. Using the Sanger Plasmodium databank, we were able to demonstrate significant homology between the MOG protein (NP_034944) and the CIR protein (PC500044.00.0). Five different regions presented some similarity when the two proteins were aligned (Fig. S1A). One of these five regions exactly matched the MOG_35-55_ peptide (Fig. S1B). However, no cross-reactivity was observed when this peptide (CIR_180-198_) was used either to induce EAE in naïve animals or to treat MOG_35-55_ immunized animals (data not shown). However, we cannot exclude the possibility of polyclonal expansion of lymphocytes promoted by the malaria infection, which may enhance MOG_35-55_ specific T regs and thereby cause the phenomena described herein, albeit in smaller numbers. Nevertheless, our data provide strong evidence that once activated by the TCR or during polyclonal expansion, T regs may exert their suppression in a completely non-specific manner. Indeed, Thornton and Shevach have proposed this non-specific suppressor mechanism using an *in vitro* model system [39]. Moreover, the regulatory functions of these T regs are probably due to their expression and release of inhibitory cytokines such as TGF-β and IL-10 in a standard suppressive manner.

In the present study we describe for the first time the ability of *Plasmodium* infection to interfere with the EAE autoimmune response. Our findings indicate that the T reg cells generated during *P. chabaudi* infection act in an apparently non-specific manner to control the proinflammatory response, mainly via increased expression of IL-10 and TGF-β_1_.

## Supporting Information

Figure S1 (**A**) Amino acid sequence alignment of the MOG (black) and CIR proteins (http://www.ebi.ac.uk: Accession number: needle-20100715-1546033194.output) (red). Green symbols indicate identical amino acids; yellow symbols represent conservative changes and blue symbols represent semi-conservative changes. (**B**) Sequence comparison of the MOG_35-55_ and CIR_180-198_ peptides.
(TIF)
