# Supplementary figures and images for: Regulatory T Cell Induction during Plasmodium chabaudi Infection Modifies the Clinical Course of Experimental Autoimmune Encephalomyelitis
Source: PLoS One. 2011 Mar 25;6(3):e17849. doi: 10.1371/journal.pone.0017849 (PMC3064572; doi:10.1371/journal.pone.0017849)

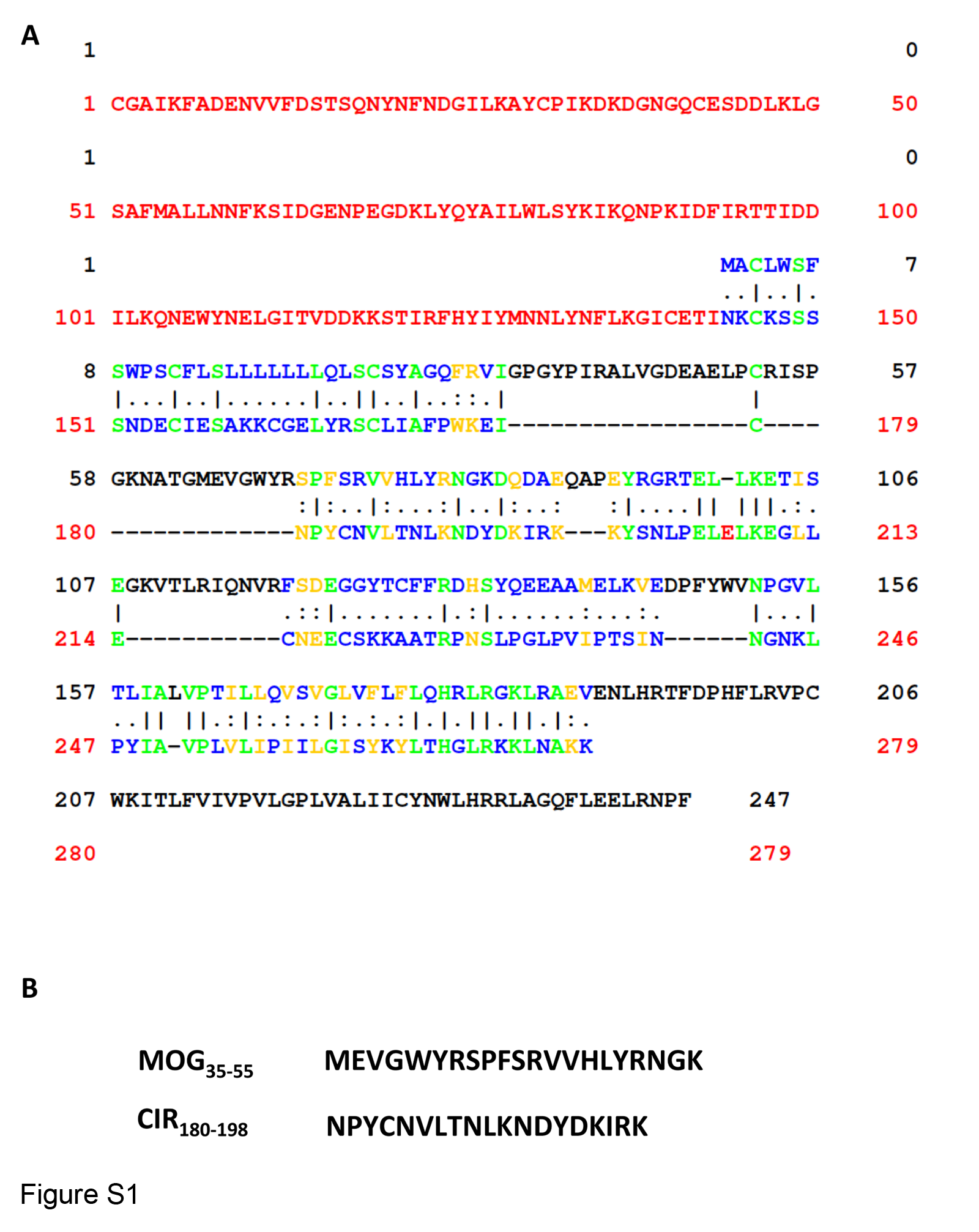

Supplement: Figure S1 — (A) Amino acid sequence alignment of the MOG (black) and CIR proteins (http://www.ebi.ac.uk: Accession number: needle-20100715-1546033194.output) (red). Green symbols indicate identical amino acids; yellow symbols represent conservative changes and blue symbols represent semi-conservative changes. (B) Sequence comparison of the MOG35-55 and CIR180-198 peptides. (TIF) [file pone.0017849.s001.tif]
